# Graph-Based Symbolic Technique and Its Application in the Frequency Response Bound Analysis of Analog Integrated Circuits

**DOI:** 10.1155/2014/202371

**Published:** 2014-07-17

**Authors:** E. Tlelo-Cuautle, S. Rodriguez-Chavez, A. A. Palma-Rodriguez

**Affiliations:** INAOE, 72840 Tonantzintla, Puebla, PUE, Mexico

## Abstract

A new graph-based symbolic technique (GBST) for deriving exact analytical expressions like the transfer function *H*(*s*) of an analog integrated circuit (IC), is introduced herein. The derived *H*(*s*) of a given analog IC is used to compute the frequency response bounds (maximum and minimum) associated to the magnitude and phase of *H*(*s*), subject to some ranges of process variational parameters, and by performing nonlinear constrained optimization. Our simulations demonstrate the usefulness of the new GBST for deriving the exact symbolic expression for *H*(*s*), and the last section highlights the good agreement between the frequency response bounds computed by our variational analysis approach versus traditional Monte Carlo simulations. As a conclusion, performing variational analysis using our proposed GBST for computing the frequency response bounds of analog ICs, shows a gain in computing time of 100x for a differential circuit topology and 50x for a 3-stage amplifier, compared to traditional Monte Carlo simulations.

## 1. Introduction

With the downscaling of the integrated circuit (IC) technology, nanometer circuit designs become more and more sensitive to process variations [1, 2], which are produced by fluctuations at the moment of manufacturing, and have been continuously increasing in relative magnitude as IC technology continues to scale to 45 nm and below. On the one hand, IC designers usually perform repeated Monte Carlo (MC) simulations to predict variations, leading to an expensive computational cost, while the main goal is computing the bounds of a given performance by varying the value of some parameters under certain percentage. On the other hand, to mitigate this drawback on performing repeated MC simulations, performance bound methods emerged as attractive techniques for variational analysis of analog ICs under parameter variations [3–7]. However, although those bound methods are quite efficient computing the lower and upper bounds of the magnitude and phase of a transfer function *H*(*s*), systematic methods have not been proposed yet to obtain the variational symbolic expression for *H*(*s*). Henceforth, this paper introduces a performance bound technique that derives the exact analytical *H*(*s*) of a linear (linearized) analog IC, as CMOS amplifiers, by applying a new graph-based symbolic technique (GBST), which is described in the next section. Afterwards, the frequency response bounds, for the magnitude and phase for *H*(*s*) of three amplifiers, are obtained by applying nonlinear constrained optimization.

## 2. Graph-Based Symbolic Technique

Symbolic analysis of analog circuits has been demonstrated to be useful for deriving analytical expressions such as *H*(*s*) [8–14]. An important contribution on the development of symbolic analysis techniques was the introduction of determinant decision diagrams (DDDs) [8, 9], whose advantage is computing symbolic expressions sharing many subexpressions [10, 11]. However, it was not realized for deriving exact analytical expressions for CMOS amplifiers [15]. That way, we introduce herein a new graph-based symbolic technique (GBST) for deriving exact symbolic expressions of analog ICs. Our proposed GBST exploits DDD concepts and has the advantage of being compact and unique as well.

### 2.1. Simple Case: Symbolic Determinant without Node Reuse

**Figure 1:**
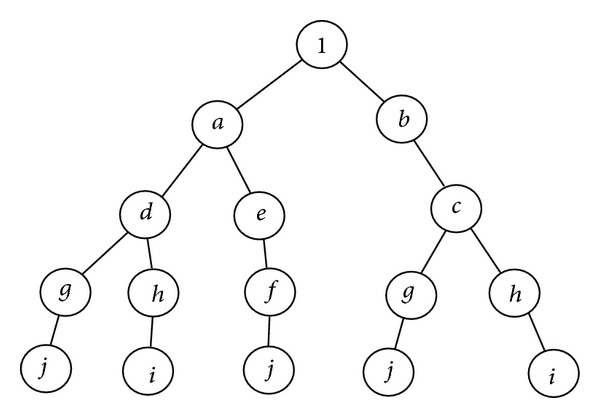
Graph representation of (1).

Let us consider the determinant given by (1) [9], of size *n* × *n*. By applying GBST, the graph representation is built in a depth-first search (DFS) fashion, while one expects having paths of *n* + 1 levels. Every element in the graph corresponds to a nonzero entry in *M*. In this manner, one obtains the graph shown in Figure 1, where applying the rule of signs from Cramer's rule (see (2)) does the assignation of signs to each node. A path is eliminated if a zero entry in the nodal admittance matrix is found. Consider

(1) $$\begin{array}{c} \left|M\right|=\left|\begin{array}{cccc} a & b & 0 & 0\\ c & d & e & 0\\ 0 & f & g & h\\ 0 & 0 & i & j \end{array}\right|=adgj-adhi-aefj-bcgj+bchi, \end{array}$$

(2) $$\begin{array}{c} \mathrm{sign}={\left(-1\right)}^{\text{row}+\text{col}}. \end{array}$$

A tree in which arithmetic operations are encoded in the depth of the tree nodes represents the graph. That is, different depth implies multiplication and equal depth implies addition. This leads us to derive the expression:

(3) $$\begin{array}{c} \left|M\right|=a\left[d\left(gj-hi\right)+e\left(-fj\right)\right]+b\left[c\left(-gj+hi\right)\right]. \end{array}$$

### 2.2. Advanced Case: Symbolic Determinant with Node Reuse

In (3), the determinant is expressed as a sequence of products [8, 9], because the graph shown in Figure 1 does not reuse node information, while having many repetitive terms that correspond to the repeated minors *gj* and *hi*. For this case, the smallest the matrix minor, the highest the repetition rate. Therefore, our main idea is reusing the information of those repeated nodes. For example: *gj*, *hi*, and *fj* are product terms of 2 × 2 minors in (1). In this manner, there are five two-nodded subgraphs with vertex sets *V*_1_ = {*g*, *j*}, *V*_2_ = {*h*, *i*}, *V*_3_ = {*f*, *j*}, *V*_4_ = {*g*, *j*}, and *V*_5_ = {*h*, *i*}. As one sees, edges *E*_1_ = {*g*, *j*} and *E*_4_ = {*g*, *j*} are equivalents, so that subgraphs *G*_1_ = {*V*_1_, *E*_1_} and *G*_4_ = {*V*_4_, *E*_4_} carry the same information. In this case, node reuse is possible, and one just needs to identify node ancestors; for example, the subgraphs *G*_1_ = *G*_4_ and *G*_3_ = {*V*_3_, *E*_3_} have the node ancestors: *g* for *G*_1_ = *G*_4_ and *f* for *G*_3_. Recall that each node is linked to a nonzero matrix entry; that is, *A*_row,col_ ≠ 0. Node *f* is in turn a representation of *A*_3,2_ and *g* of *A*_3,3_.

Extending node reuse in the whole graph, the first obvious consequence is that there are no repeated nodes; in other words, for a matrix *A* with *nz* nonzero entries, there are *nz* nodes.

### 2.3. The Advanced Case in Detail

To derive the symbolic expression with node reuse, three different data structures are required. The first and most obvious is the node structure that contains the following:

- node name: a unique name for each node, assigned as an index number;
- terms: an array containing the index and sign of the element;
- column: the column of the nonzero entry where the node belongs to;
- descendants: an array of node pointers linking to the descendants of the current node in the graph structure.

The second data structure is a graph type with the fields:

- graph name: a unique name for the current graph. It is possible to have many different graphs; for example, to compute the transfer function *H*(*s*), two graphs are required: one for deriving the numerator and the other for the denominator [9];
- matrix size: the size of the square matrix;
- root node: it is a trivial node with term value equal to 1 (see Figure 1) and row and column equal to zero. When multiple graphs are constructed (during factorization), the root node can be any of the *nz* nodes;
- visited columns: when traversing a graph to represent the determinant, the column of a visited node is appended to this array.

The third structure stores matrix elements and the independent vector.

**Figure 2:**
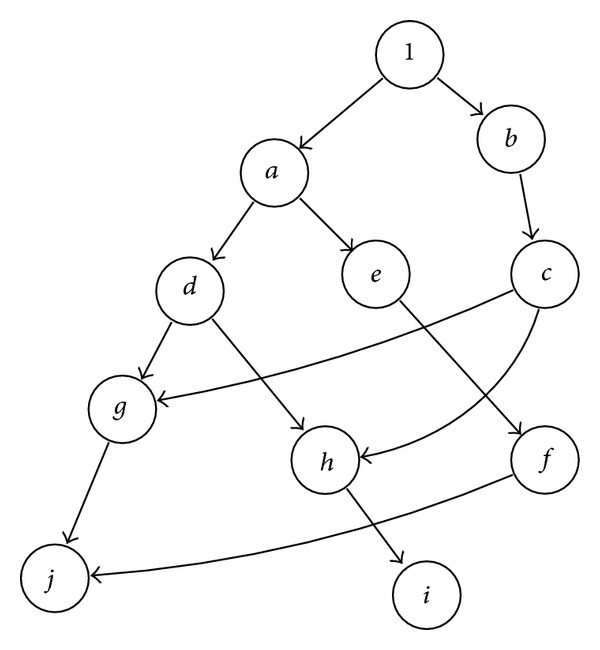
Determinant graph with node reuse.

From these structures, *nz* + 1 nodes are created. The row and column fields are used to evaluate the sign by (2) as well as to determine which nodes are to be skipped. The graph is generated starting with a trivial node named 0 with term value of 1; then the multiplication of terms is codified as the depth in the graph, and nodes are linked accordingly. The algorithm to build the graph structure for the representation of |*A*| is sketched by Algorithm 1.  Algorithm 2 computes the expression for the determinant. That way, the graph associated to the determinant in (1) is shown in Figure 2.

### 2.4. Symbol Factorization

Factorization takes place by executing Algorithm 3. The result is the expression as a polynomial that is represented by an array of sum of products with one entry for each power of symbol *w*.

**Figure 3:**
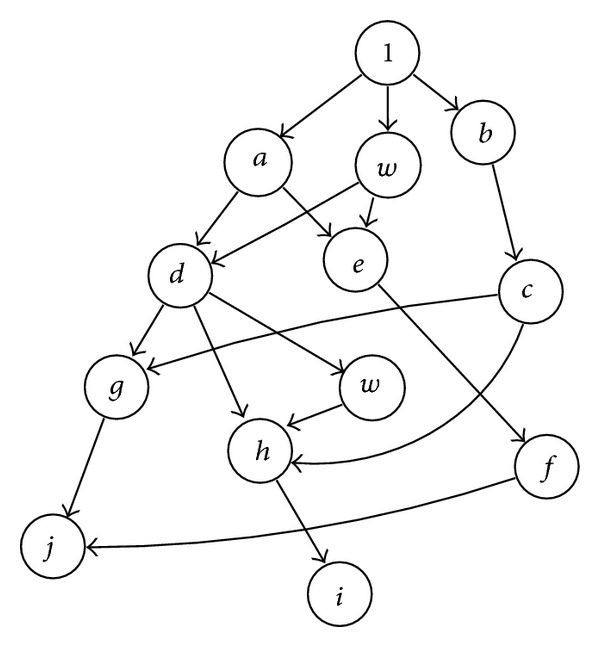
Expanded graph.

Let us consider matrix *M* in (4). To derive the polynomial form as powers of *w*, the associated graph is shown in Figure 3, and the polynomial is given by (5). Consider

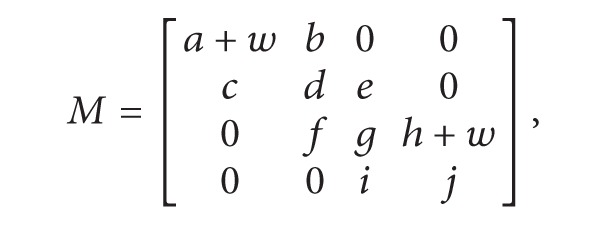
(4)

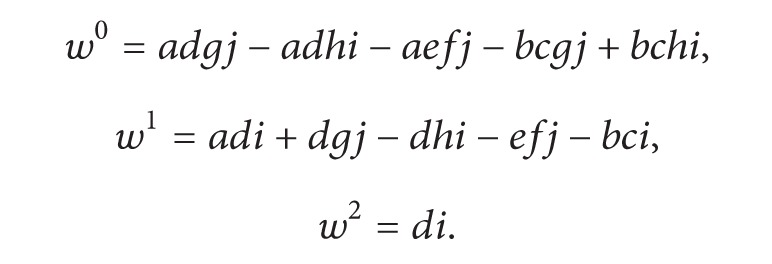
(5)


### 2.5. Applying the Graph-Based Symbolic Technique

Our proposed GBST starts off with an SPICE-like netlist as input. The allowed circuit elements are R, C, L, V, I, E, G, and M, being resistor, capacitor, inductor, independent voltage source, independent current source, voltage-controlled voltage source, voltage-controlled current source, and MOSFET. When formulating the equations, the symbol name is taken exactly as specified in the netlist, for example, R_name, C_name, M_name, and so forth. Their corresponding small-signal model based on controlled sources substitutes all active elements. In turn, controlled sources are modeled with combinations of norator and nullator in order to make use of the extensive studies on analysis of Nullor based circuits given in [9, 11, 14, 15]. The guidelines for obtaining the nodal admittance matrix by applying nodal analysis are summarized in [11]. Afterwards, applying our proposed graph-based symbolic technique derives the solution. In this manner, the computing information on the solutions for the common source amplifier, differential pair amplifier, three stages uncompensated OTA [15], recycled folded cascode OTA [16], and 741 opamp is listed in Table 1. Using the values computed by HSPICE, in order to verify correctness, does the numerical evaluation of the derived symbolic expression.

**Figure 4:**
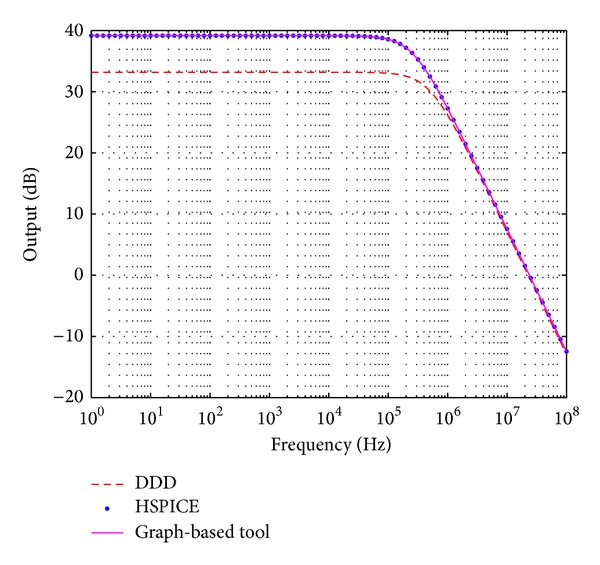
*H*(*s*) comparison among DDD [3] (dashed), HSPICE (dots), and our graph-based symbolic technique (solid) for the differential pair topology.

To demonstrate the suitability of the new graph-based symbolic technique (GBST), we performed a comparison with HSPICE and the DDD symbolic tool [3], as shown in Figure 4. As one sees, GBST is in good agreement with the numerical response computed by SPICE, while the DDD technique has an error around 10%. That way, GBST is applied herein to derive the exact analytical expression *H*(*s*) of CMOS amplifiers. The derived *H*(*s*) is used to perform variational analysis, in order to compute the frequency response bounds (maximum and minimum) of the magnitude and phase from *H*(*s*), subject to some ranges of process variational parameters by performing nonlinear constrained optimization.

## 3. Constrained Optimization

To highlight the appropriateness of applying the new GBST in variational analysis for finding the lower and upper performance bounds for the magnitude and phase of an amplifier, we formulate a nonlinear constrained optimization problem. That way, we start from a transfer function, whose coefficients (e.g., *a*_*i*_(*p*_1_,…, *p*_*m*_) and *b*_*j*_(*p*_1_,…, *p*_*n*_)) are obtained by our proposed graph-based symbolic technique in an* s*-expanded form; that is,

(6) $$\begin{array}{c} H\left(\omega \right)=\frac{\sum_{i=0}^{m}a_{i}\left(p_{1},\ldots ,p_{m}\right)s^{i}}{\sum_{j=0}^{n}b_{j}\left(p_{1},\ldots ,p_{n}\right)s^{j}}. \end{array}$$

Notice that *H*(*s*, *p*_1_,…, *p*_*m*_) is a nonlinear function of *p*_1_,…, *p*_*m*_. Furthermore, each parameter *p*_*i*_ is a random variable with a variational range.

For *s* = *jω*, the nominal transfer function becomes

(7) $$\begin{array}{c} H\left(s\right)=H\left(j\omega \right)=H\left(j\omega \right)e^{j\theta (\omega )}. \end{array}$$

**Figure 5:**
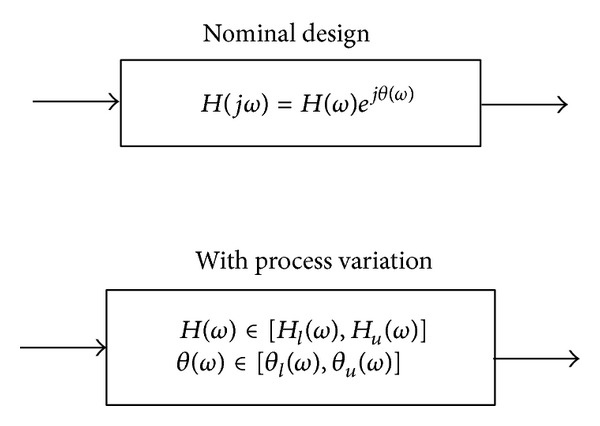
Transfer function description including parameter variations.

From it, one can obtain a variational transfer function with bounded magnitude and phase regions, which is described by [3]

(8) $$\begin{array}{c} H_{l}\left(\omega \right)\leq H\left(\omega \right)\leq H_{u}\left(\omega \right),\\ \theta_{l}\left(\omega \right)\leq \theta \left(\omega \right)\leq \theta_{u}\left(\omega \right), \end{array}$$

where *H*_*l*_(*ω*) and *H*_*u*_(*ω*) are the lower and upper bounds of the magnitude, respectively, and *θ*_*l*_(*ω*) and *θ*_*u*_(*ω*) are the lower and upper bounds of the phase. The evaluation of (7) gives a complex valued result, where the magnitude *H*(*ω*) = |*H*(*jω*)| and the phase angle *θ*(*ω*) = *∠H*(*jω*) are real values. The goal is to find the bounds of the magnitude and phase for *H*(*jω*), such that one can obtain (8). Henceforth, in the presence of process variations, the signal is perturbed from its nominal behavior, and it is usually bounded between its minimum and maximum limits, as sketched in Figure 5.

For instance, using the lower bound of the magnitude response |*H*(*jω*)| at frequency *ω*, then the objective function is minimized and described by

(9) $$\begin{array}{c} \text{minimize}\;\left|H\left(j\omega ,x\right)\right|\\ \text{subject}\;\text{to}\;\;x_{l}\leq x\leq x_{u}. \end{array}$$

In (9) *x* = [*p*_1_,…, *p*_*m*_] represents the circuit parameters variable vector, subject to the optimization constraints [*x*_*l*_, *x*_*u*_]. In IC design foundries and cell library vendors supply the constraints.  Algorithm 4 summarizes this process.

### 3.1. A Simple Example in MATLAB

**Figure 6:**
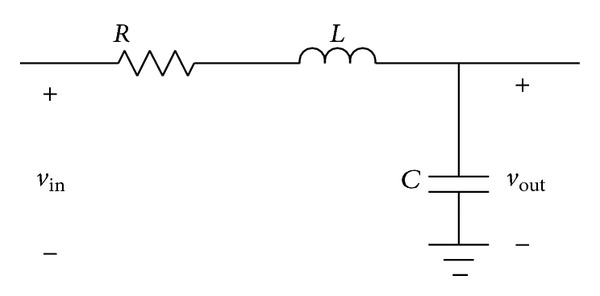
RLC circuit.

Let us consider Figure 6. In the frequency domain, capacitors and inductors are analyzed as complex impedances, and then the transfer function is given by

(10) $$\begin{array}{c} H\left(j\omega \right)=\frac{V_{\text{out}}}{V_{\text{in}}}=\frac{1/j\omega C}{R+j\omega L+\left(1/j\omega C\right)}. \end{array}$$

**Figure 7:**
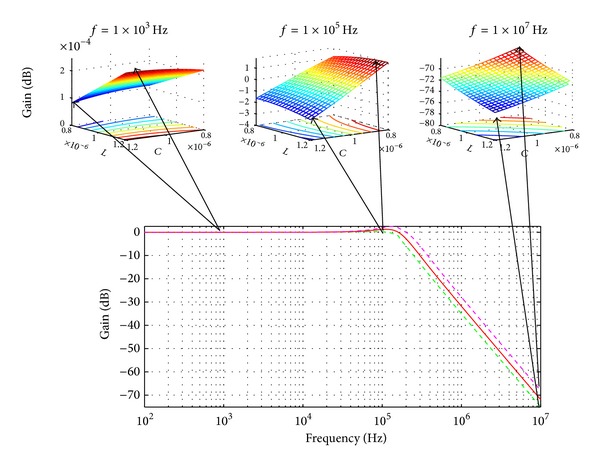
Frequency response of the RLC circuit [3]. Solid curve is the magnitude response with nominal parameters. The dashed curves are the lower and upper bounds due to variations. The three surfaces at the top have *L* and *C* as *x*-axis and *y*-axis, respectively, and *z*-axis shows the magnitude variations at three sampling frequencies.

Assuming that *C* and *L* have variations of 20% from their nominal values *C* = 1 *μF* and *L* = 1 *μH*, then *C* ∈ [0.8,1.2]  *μF* and *L* ∈ [0.8,1.2]  *μH*. For this example, the iterative method called active set was used. As a result, three snapshots at different frequency points are shown in Figure 7.

### 3.2. Line Search Algorithm

A line search algorithm searches for a decrease in *f* in a descent direction using the Armijo rule [17, 18] for stepsize control. The steplength is chosen to minimize *f* along the ray {*x* + *λ*Δ*x*∣*λ* ≥ 0}, where Δ*x* is called the* descent direction* and the point *x* + *λ*Δ*x* is called *x*_+_, or even to just reduce *f* “*enough*” [19]. That way, given the current point *x*_*c*_ and descent direction Δ*x*, it looks for *λ*, such that

(11) $$\begin{array}{c} f\left(x_{c}+\lambda \Delta x\right)\leq f\left(x_{c}\right). \end{array}$$

**Figure 8:**
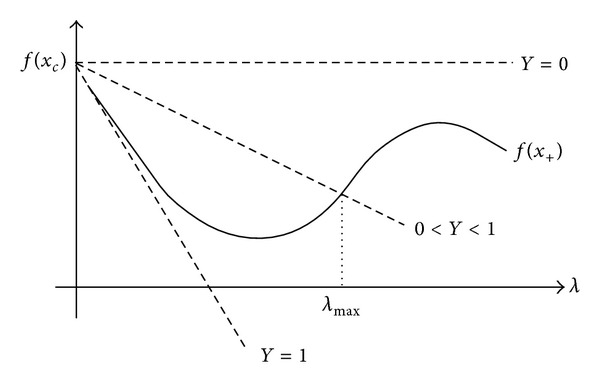
Line search representation.

However, if the decreasing achieved by this inequality for some *λ* is too small, it is not possible to guarantee convergence to a local minimum. So in order to avoid this issue, *λ* must satisfy* Armijo rule* (*sufficient decrease*) given by

(12) $$\begin{array}{c} f\left(x_{c}+\lambda \Delta x\right)\leq f\left(x_{c}\right)+\lambda \gamma \nabla f{\left(x_{c}\right)}^{T}\Delta x, \end{array}$$

where *γ* ∈ (0,1) [20]. This is shown in Figure 8.

In Figure 8, the interval where (12) is accomplished is [0, *λ*_max⁡_]. We can rewrite this by (13) where the Armijo condition [18] to accept a trial point *x*_+_ is given by (14), and *M* is an integer greater than zero. If *x*_+_ is rejected, the steplength is redefined by (15), where λ* minimizes a quadratic model. This strategy of repeatedly testing sufficient decrease and reducing the stepsize if the test fails is called backtracking. The projected gradient (PGRAD) and spectral projected gradient (SPG) methods used herein for variational analysis of CMOS amplifiers are based on backtracking line search. Consider

(13) $$\begin{array}{c} f\left(x_{+}\right)\leq f\left(x_{c}\right)+\gamma \nabla f{\left(x_{c}\right)}^{T}\left(x_{+}-x_{c}\right), \end{array}$$

(14) $$\begin{array}{c} f\left(x_{+}\right)\leq \underset{0\leq j\leq \min\left\{c,M-1\right\}}{\max}f\left(x_{c-j}\right)+\gamma \nabla f{\left(x_{c}\right)}^{T}\left(x_{+}-x_{c}\right), \end{array}$$

(15) $$\begin{array}{c} \lambda =\max\left\{\sigma_{1}\lambda ,\min\left\{\sigma_{2}\lambda ,\lambda^{*}\right\}\right\}. \end{array}$$

### 3.3. Projected Gradient Method

The PGRAD method is described in [21]. It is the natural extension of the* steepest descent algorithm* [20], used in unconstrained optimization to bound constrained problems.

Given a current iterate *x*_*c*_, the new iterate is

(16) $$\begin{array}{c} x_{+}=P\left(x_{c}-\lambda \nabla f\left(x_{c}\right)\right), \end{array}$$

where the gradient ∇*f*(*x*) is defined by

(17) ∇f(x)=(∂f(x)∂x1,…,∂f(x)∂xn)for x=(x1,…,xn).

In (17), *λ* is a steplength parameter given by the Armijo rule, where one searches on a ray from *x*_*c*_ in a direction where *f* is locally decreasing [18]. Besides, in order to implement a line search scheme, one must specify what sufficient decrease means. Therefore, for bound constrained problems the* sufficient decrease* condition for line search is defined by

(18) $$\begin{array}{c} f\left(x\left(\lambda \right)\right)-f\left(x\right)\leq \frac{-\alpha }{\lambda }{||x-x\left(\lambda \right)||}^{2}, \end{array}$$

where

(19) $$\begin{array}{c} x\left(\lambda \right)=P\left(x-\lambda \nabla f\left(x\right)\right)\;\text{for}\;\;\lambda \geq 0. \end{array}$$

In this case *λ* = *β* ^*m*^, where *β* ∈ (0,1) and *m* ≥ 0, which is the smallest nonnegative integer such that there is* sufficient decrease* in *f*. For the end condition, it is necessary to define the active and inactive sets. The set of constraints is called* feasible set* (Ω), and a point in this set is a* feasible point*. Because the* feasible set* is compact, there is always a solution for this minimization problem. The *i*th constraint is* active* at *x* ∈ Ω if either *x*_*i*_ = *L*_*i*_ or *x*_*i*_ = *U*_*i*_. If the *i*th constraint is not* active*, it is called* inactive*. Therefore, one can write *A*(*x*) and *I*(*x*) for the active and inactive sets, respectively. An* active/inactive set* is the set of indexes *i* such that the *i*th constraint is active/inactive. In this manner,

(20) $$\begin{array}{c} r_{0}=||x_{0}-x_{0}\left(1\right)||. \end{array}$$

The stop criterion is given by (21), where *τ*_*r*_ and *τ*_*a*_ are relative and absolute tolerances. Consider

(21) $$\begin{array}{c} ||x-x\left(1\right)||\leq \tau_{a}+\tau_{r}r_{0}. \end{array}$$

This optimization method is shown in Algorithm 5. It starts with *x*_0_ ∈ Ω and uses a sufficient decrease parameter *γ* ∈ (0,1) and safeguarding parameters 0 ≤ *σ*_1_ ≤ *σ*_2_ ≤ 1. Initially, *α*_0_ = 1/||*f*(*x*_0_)||^2^. Given *x*_*c*_ and *α*_*c*_ ≥ 0 the algorithm shows how to obtain *x*_*c*+1_ and *α*_*k*+1_ and when to stop the optimization engine.

### 3.4. Spectral Projected Gradient Method

The SPG method improves choosing the steplength, which greatly speeds up the convergence of gradient methods [22]. Unlike the projected gradient method, the spectral projected gradient one [21] is more related to the quasi-Newton family [17]. The main idea behind the spectral choice of steplength is that the steepest descent method is very slow but it can be accelerated taking, instead of the stepsize that comes from the minimization of the function along the gradient of the current iteration, the one that comes from the one-dimensional minimization at the previous step.

The point in the first iteration of this method should be a feasible point; that is, the algorithm starts with *x*_0_ ∈ Ω and uses an integer *M* ≥ 1, a small parameter *α*_min⁡_ ≥ 0, a large parameter *α*_max⁡_ ≥ *α*_min⁡_, a sufficient decrease parameter *γ* ∈ (0,1), and safeguarding parameters 0 ≤ *σ*_1_ ≤ *σ*_2_ ≤ 1. Consider ||*P*(*x*_*c*_ − ∇*f*(*x*_*c*_)) − *x*_*c*_|| = 0.

The method is shown in Algorithm 6, where *λ*_new_ uses one-dimensional quadratic interpolation and it is safeguarded taking *λ* ← *λ*/2 when the minimum of the one-dimensional quadratic lies outside [0.1,0.9*λ*]. The line search condition in step 6 guarantees that the sequence {*x*_*c*_} remains in Ω_0_ = {*x* ∈ Ω : *f*(*x*) ≤ *f*(*x*_0_)}.

### 3.5. Remarks

The projected gradient and spectral projected gradient methods start at *x*_0_ ∈ Ω and use as search direction the internal projected gradient direction. In case of rejection of the first trial point, the next ones are computed along the same line. Also, for both methods, the calculation of *λ*_new_ uses a one-dimensional quadratic interpolation. Both algorithms involve a projection on the convex set Ω, a function evaluation *f*(*x*), and a gradient evaluation per iteration ∇*f*(*x*).

## 4. Simulation Results

**Figure 9:**
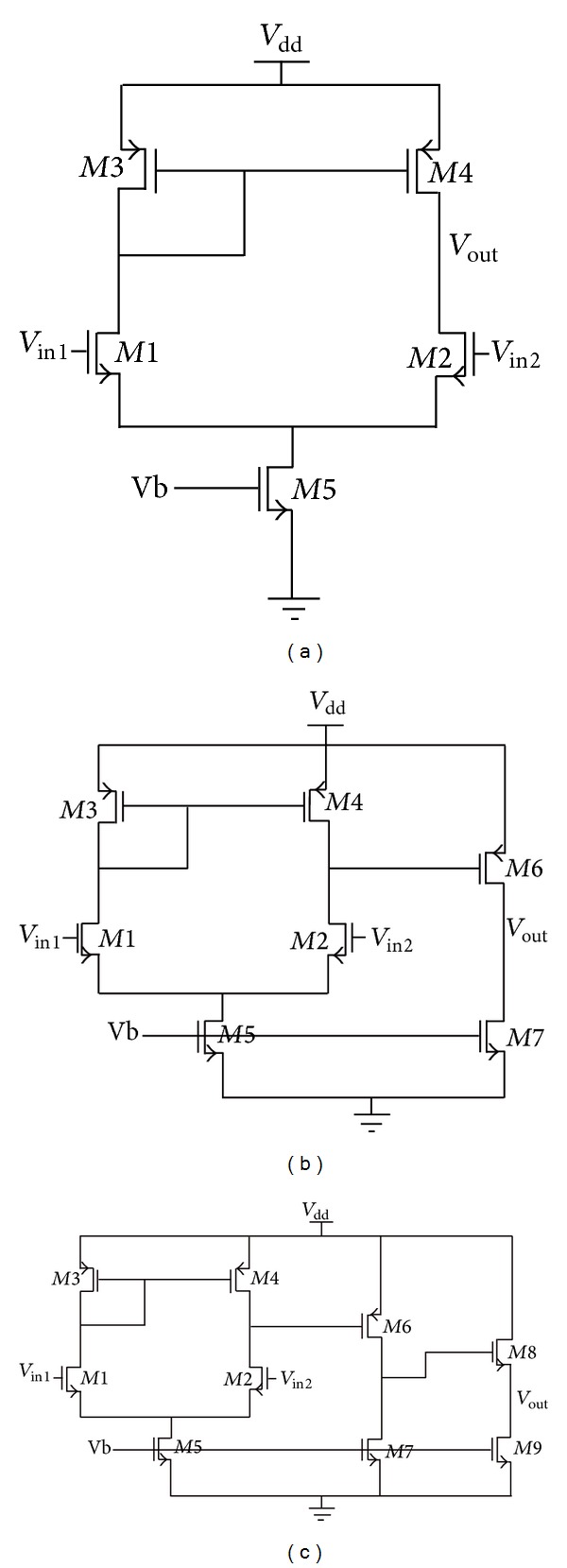
Circuits used for the variational analysis: (a) differential pair, (b) 2-stage OTA, and (c) 3-stage OTA.

The analog ICs for testing the variational methods PGRAD and SPG that use *H*(*s*) derived by the new GBST are shown in Figure 9. That way, after deriving the transfer function by applying the new GBST, and after setting the desired frequencies, the PGRAD and SPG methods are applied to find the magnitude and phase bounds for *H*(*s*).

**Figure 10:**
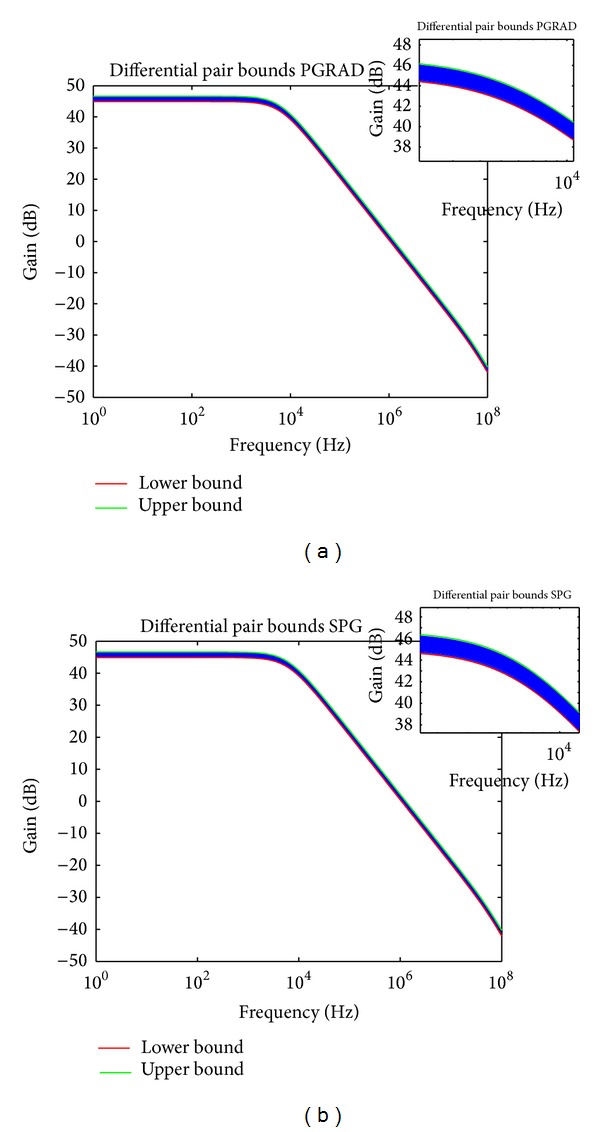
Bounds for the differential pair applying (a) PGRAD and (b) SPG.

**Figure 11:**
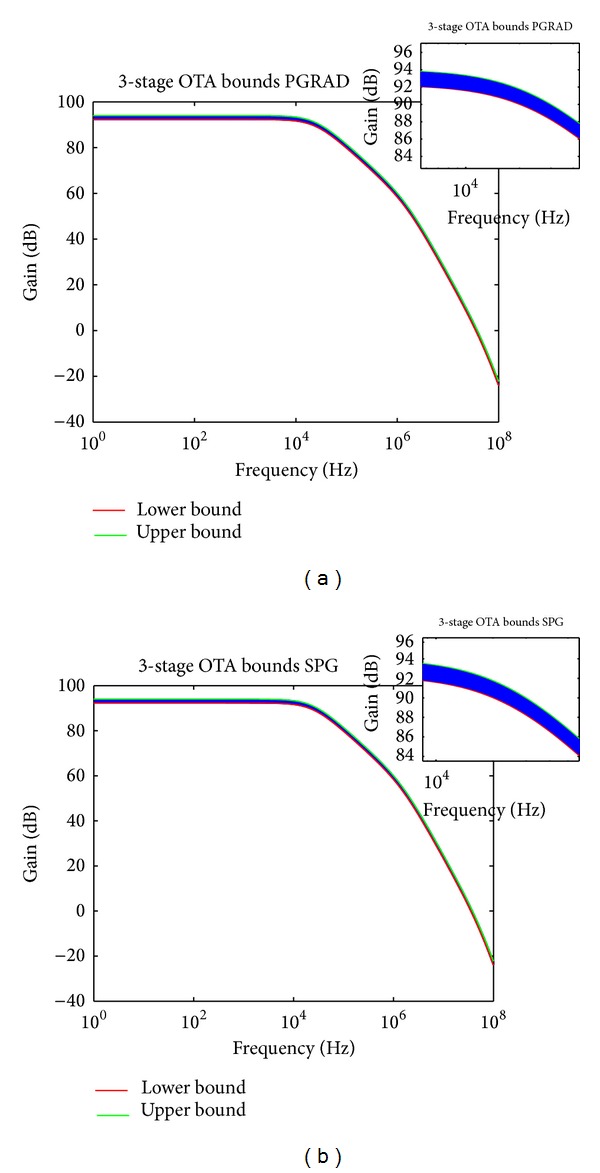
Bounds for the 3-stage OTA applying (a) PGRAD and (b) SPG.

Both the PGRAD and SPG methods were programmed in C and compiled in an Ubuntu Linux environment with the GNU C compiler gcc-4.6.1, with 4 GB RAM in an Intel Core i3. The performance bounds results for the differential pair are shown in Figure 10 and for the 3-stage OTA in Figure 11. The lines in blue are those corresponding to Monte Carlo simulations, which are well bounded by the bounds computed by the variational methods PGRAD and SPG.  Table 2 summarizes the results where it can be appreciated that both variational methods show better times than by performing repeated Monte Carlo simulations when using HSPICE.

## 5. Conclusions

A new graph-based symbolic technique (GBST) for deriving exact analytical expressions for analog ICs, such as *H*(*s*), and whose symbolic expressions are used to perform variational analysis to obtain performance frequency bounds has been introduced. The variational analysis needs as input the *H*(*s*) computed by applying our proposed GBST, and it was based on nonlinear constrained optimization using two line search methods, namely, projected gradient (PGRAD) and spectral projected gradient (SPG).

It was demonstrated that the bound analysis based on GBST is suitable for analog ICs. In addition, the time computation of PGRAD and SPG for obtaining the frequency response bounds of analog ICs was improved with respect to HSPICE, when performing repeated Monte Carlo simulations. As shown in the last section, computing the frequency response bounds by using GBST showed a gain of 100x for the differential pair and 50x for the 3-stage amplifier, compared to repeated Monte Carlo simulations, thus justifying the usefulness of using GBST.

## Figures and Tables

**Algorithm 1:**
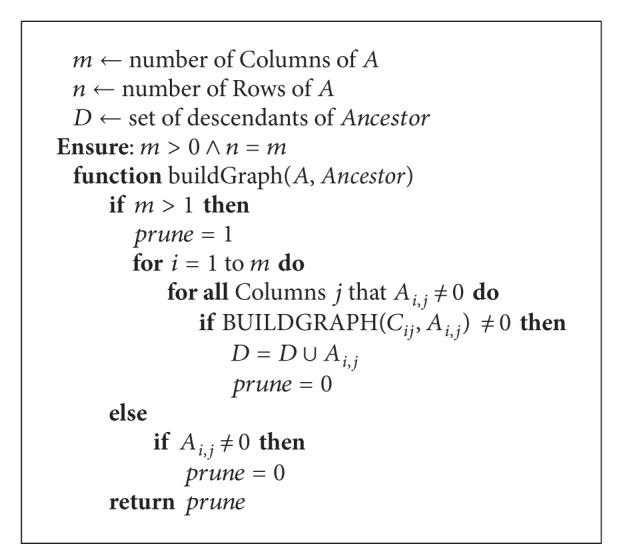
Build graph (*A*(*i*, *j*), *Ancestor*).

**Algorithm 2:**
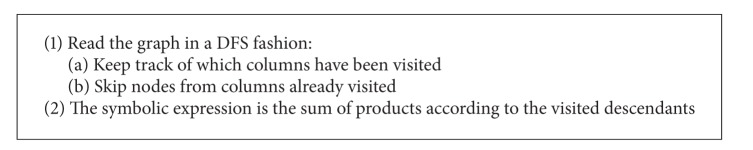
Symbolic determinant from a graph.

**Algorithm 3:**
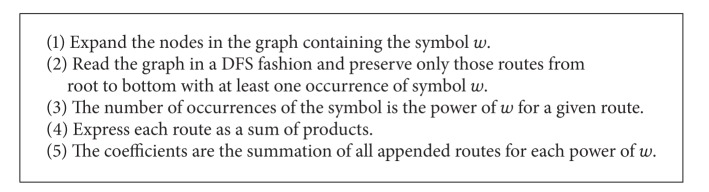
Graph to polynomial form.

**Algorithm 4:**
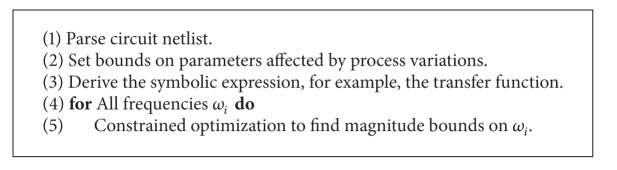
Calculation of frequency response bounds using our graph-based symbolic technique from Section 2 and constrained optimization.

**Algorithm 5:**
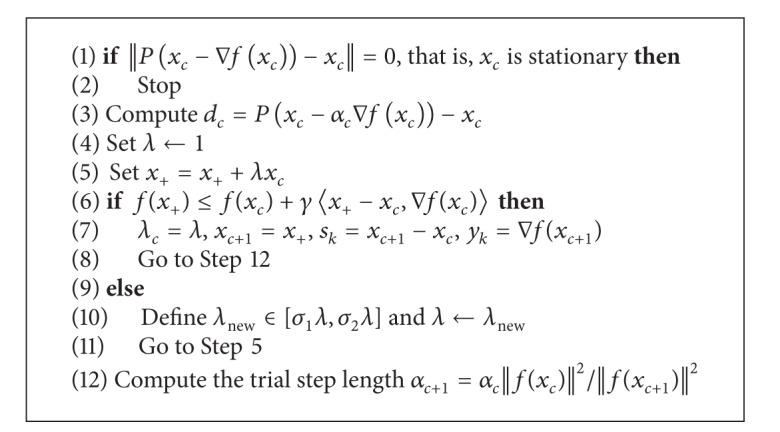
Projected gradient method.

**Algorithm 6:**
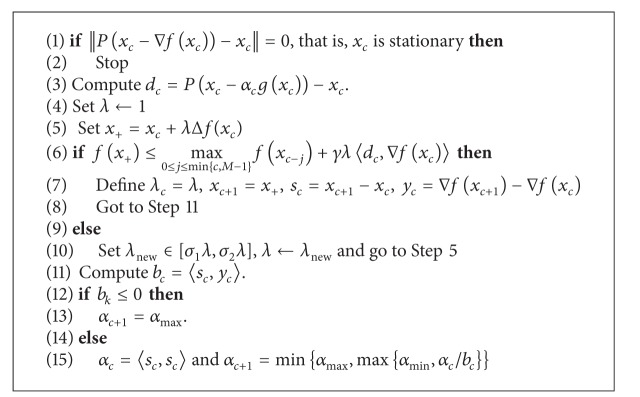
Spectral projected gradient method.

**Table 1:** Symbolic formulation and numerical evaluation of *D*(*s*), *N*(*s*), and*H*(*s*) by applying the new GBST.

Circuit features			Computer time (seconds)			
Circuit	Elements	Nodes	Equations	*D*(*s*)	*N*(*s*)	*H*(*s*)
Differential pair	35	26	1.1235	0.122	0.1464	1.4895
RFC OTA [16]	106	56	1.6603	0.201	0.1869	2.2633
LV Amp [15]	33	18	2.35	0.058	0.0464	2.4544
Common source	8	6	0.8581	0.041	0.0205	0.9811
741	112	77	0.5123	1.37	0.822	2.7043

**Table 2:** Time comparison between the variational methods and HSPICE.

Circuit	PGRAD	SPG	HSPICE
Differential pair	15.312 ms	16.363 ms	1.716 s
2-stage OTA	44.559 ms	91.936 ms	81.65 ms
3-stage OTA	38.315 ms	39.233 ms	2.163 s
